# Ethnic Variations in the Prevalence of Diabetic Retinopathy in People with Diabetes Attending Screening in the United Kingdom (DRIVE UK)

**DOI:** 10.1371/journal.pone.0032182

**Published:** 2012-03-08

**Authors:** Sobha Sivaprasad, Bhaskar Gupta, Martin C. Gulliford, Hiten Dodhia, Moin Mohamed, Dinesh Nagi, Jennifer R. Evans

**Affiliations:** 1 King's College Hospital NHS Foundation Trust, London, United Kingdom; 2 Division of Health and Social Care Research, King's College London, London, United Kingdom; 3 Lambeth NHS Primary Care Trust, London, United Kingdom; 4 St Thomas Hospital NHS Foundation Trust, London, United Kingdom; 5 Mid Yorkshire NHS Trust, Wakefield, West Yorkshire, United Kingdom; 6 London School of Hygiene and Tropical Medicine, London, United Kingdom; Fundación para la Prevención y el Control de las Enfermedades Crónicas No Transmisibles en América Latina (FunPRECAL), Argentina

## Abstract

**Aims:**

To compare the prevalence of diabetic retinopathy (DR) in people of various ethnic groups with diabetes in the United Kingdom (UK).

**Methods:**

The **D**iabetic **R**etinopathy **I**n **V**arious **E**thnic groups in UK (DRIVE UK) Study is a cross-sectional study on the ethnic variations of the prevalence of DR and visual impairment in two multi-racial cohorts in the UK. People on the diabetes register in West Yorkshire and South East London who were screened, treated or monitored between April 2008 to July 2009 (London) or August 2009 (West Yorkshire) were included in the study. Data included age, sex, ethnic group, type of diabetes, presenting visual acuity and the results of grading of diabetic retinopathy. Prevalence estimates for the ethnic groups were age-standardised to the white European population for comparison purposes.

**Results:**

Out of 57,144 people on the two diabetic registers, data were available on 50,285 individuals (88.0%), of these 3,323 had type 1 and 46,962 had type 2 diabetes. In type 2 diabetes, the prevalence of any DR was 38.0% (95% confidence interval(CI) 37.4% to 38.5%) in white Europeans compared to 52.4% (51.2% to 53.6%) in African/Afro-Caribbeans and 42.3% (40.3% to 44.2%) in South Asians. Similarly, sight threatening DR was also significantly more prevalent in Afro-Caribbeans (11.5%, 95% CI 10.7% to 12.3%) and South Asians (10.3%, 9.0% to 11.5%) compared to white Europeans (5.5%, 5.3% to 5.8%). Differences observed in Type 1 diabetes did not achieve conventional levels of statistical significance, but there were lower numbers for these analyses.

**Conclusions:**

Minority ethnic communities with type 2 diabetes in the UK are more prone to diabetic retinopathy, including sight-threatening retinopathy and maculopathy compared to white Europeans.

## Introduction

Diabetic retinopathy (DR) is the most common microvascular complication of diabetes. Visual loss from diabetic retinopathy results primarily from two complications. New vessels grow on the retina; this is known as proliferative retinopathy and accounts for the majority of severe visual loss. In addition, retinal blood vessels can become permeable and cause swelling of the centre of the retina, called diabetic macular oedema. Clinically significant macular oedema is a leading cause of moderate visual loss in diabetes. Proliferative retinopathy, severe non-proliferative retinopathy and clinically significant macular oedema can be considered as sight threatening retinopathy. The established risk factors of DR include prolonged exposure to hyperglycaemia and hypertension. However, DR can progress despite optimal control of these risk factors [1].

Ethnicity is considered a complex risk factor of diabetes. Type 2 diabetes is estimated to be three to four times more common in people of Asian and African–Caribbean origin compared to white Europeans [2]. The current population in most metropolitan cities in UK is ethnically diverse [3], but differs in relative proportions of ethnic origins when compared to the US population, where contemporary comparative data on the prevalence of diabetic retinopathy in multiethnic cohorts are available [4], [5]. The healthcare system in the UK also differs from that in the US. So it is useful to obtain ethnicity specific estimates of DR in the UK to understand the impact of the changing population trends on the prevalence of DR.

The aim of this study was to compare the prevalence of diabetic retinopathy in the three main ethnic groups in the UK- white Europeans, African/Afro-Caribbeans and South Asians. In order to obtain a nationally representative sample of people with diabetes, the prevalence was estimated in two multiracial cohorts in the UK from the North and South of England- West Yorkshire and South East London.

## Methods

The study was approved by the Chair of the Research Ethics Committee at King's College Hospital NHS Foundation Trust and the London School of Hygiene and Tropical Medicine. As anonymised datasets were used, the need for individual patient consent was waived by the committee.

### Study population

Anonymised datasets on people with diabetes in the multi-racial communities in West Yorkshire and South East London were analysed in this study. The ethnic minority in West Yorkshire is mainly South Asians while Afro-Caribbeans represent the predominant minor ethnic group in South East London. People who are ascertained to have diabetes by their family practitioner are referred to the local DR screening service for annual screening as part of the nationwide DR screening programme. These digital photographic DR screening programmes in the UK are well-established and 100% of people with diabetes are offered screening and the uptake rates are at least 80% [6]. Individuals with screen- positive disease are referred to specified hospital eye services for further management so it is possible to collate the retinopathy data on all people with diabetes subject to utilization of these services. All subjects in the diabetic screening register of both these programmes were included in this study to provide a reasonably comprehensive coverage of diabetic people in the respective regions (95% In West Yorkshire and 81% in South East London).

Demographic data collected include age, sex, ethnic group and type of diabetes. Self-reported ethnicity based on UK census standard for classifying the ethnic composition of the communities were recorded at the time of screening according to the codes used in the Census 2001 and then categorised into ‘White European’, ‘African/Afro-Caribbean’, ‘South Asian’, ‘Mixed’, ‘other ethnic group’ and ‘not known’. Effort was made to obtain ethnicity data from hospital services and primary care records for missing ethnicity records within the screening programmes before the records were anonymised.

### Screening and grading of diabetic retinopathy

As part of the annual screening procedure per subject, 2-field digital photographs are taken per eye, one centred on the optic disc and the other on the macula after dilation of the pupils. Photographs undergo primary and secondary grading and, if necessary, are subjected to a final arbitration grading process according to English Diabetic Retinopathy guidance recommended by the National Screening Programme for Diabetic Retinopathy (table 1) [6], [7]. All referrals of ‘screen positive’ patients (R2, R3 and M1) were graded by retinal specialists before referral to the ophthalmology department. The retinopathy grades obtained by slit lamp biomicroscopy or indirect ophthalmoscopy were recorded for people for whom it was technically difficult to acquire retinal photographs. The records of people who were exempted from the screening programme because they are under the care of the specified hospital eye service or are blind were collated from hospital records and the registers for visually impaired.

**Table 1:** Disease grading protocol in National Guidelines on Screening for Diabetic retinopathy grading in England and Wales screening programmes.

Level	Equivalent disease severity level^9^	Clinical features
*Retinopathy*		
R0	No retinopathy	
R1	Mild and moderate non-proliferative diabetic retinopathy	Microaneurysms; retinal haemorrhages or exudates not within the definition of maculopathy
R2	Severe non-proliferative diabetic retinopathy	Venous beading/loop/reduplication;intraretinal microvascular abnormality ;multiple deep, round or blot haemorrhages
R3	Proliferative diabetic retinopathy	New vessels disc or elsewhere
*Maculopathy*		
M0		No maculopathy
M1		Exudate within 1 disc diameter of the centre of the fovea; circinate or group of exudates within the macula; retinal thickening within 1 disc diameter of the centre of the fovea; any microaneurysm or haemorrhage within 1 disc diameter of the centre of the fovea only if associated with a best visual acuity of 6/12 or worse.
*Photocoagulation*		
P0		No photocoagulation
P1		Evidence of focal or grid laser or peripheral scatter
*Unclassifiable*		
U		Unobtainable/ungradable

9 International classification proposed by American Academy of Ophthalmologists.

The number of people for whom no records were obtainable was noted, but no further details on the eye condition or ethnicity could be obtained. In addition, there may be people with very recently diagnosed diabetes (less than 12 weeks from referral as newly diagnosed diabetes) or who have only recently registered with family practices in the area, who have not been included.

We estimated the prevalence of: (a) any DR (R1, R2 and R3) (b) diabetic maculopathy (M1) (c) clinically significant macular oedema treated with laser photocoagulation (M1 P1) and (d) sight threatening diabetic retinopathy (R2, R3 or M1P1). These categories provided the closest comparison to studies that used the International classification of diabetic retinopathy scale and the Early Treatment Diabetic Retinopathy Study (ETDRS) levels [8], [9].

The data analysis was performed using Stata version 11.0 (Stata Corp., College Station, TX, USA). Data were analysed at the person level. The eye with the more severe grade of retinopathy was used in the analyses. Descriptive analyses included reporting the prevalence of the different diabetic retinopathy grades in type 1 and 2 diabetes in the three target ethnic groups. Prevalence estimates for the different ethnic groups were directly standardised to the white population which comprised the largest group. Logistic regression analyses, including terms for age (continuous), gender (male/female), diabetes (type 1/type 2), ethnic groups (white European, African/Afro-Caribbean, South Asians) and location (South London/West Yorkshire),were used to assess the independent association between factors and risk of any diabetic retinopathy, clinically significant macular oedema and proliferative diabetic retinopathy.

## Results

There were 20,878 people with known diabetes on the diabetes register of West Yorkshire region in August 2009 and 36,266 in South East London in July 2009. The total population in these areas in 2009–2010 was estimated to be 534,883 and 868,322 respectively [3]. Thus, the prevalence of diagnosed diabetes in the two study areas combined is 4.1% which is similar to the average national prevalence of diagnosed diabetes (4%). Table 2 compares the estimates of diagnosed diabetes in the study areas with the Association of Public Health Observatory model of the prevalence of diagnosed and undiagnosed diabetes [3]. This suggests that undiagnosed diabetes is highest in the Asian population.

**Table 2:** Estimated prevalence of diabetes (type 1 and type 2) in the two study areas combined in 2009–2010.

Ethnic group	Number of people estimated to be resident in two study areas combined^a^	Current study: % of population currently on the diabetic register(number of people)	YHPO model: estimated % of population with diagnosed or undiagnosed diabetes in two study areas combined^b^(number of people)
White Europeans	980,992	3.4 (33,009)	6.7 (65,603)
African-Caribbean	177,222	4.7 (8,376)	9.1 (16,151)
South Asian	81,649	4.3 (3,518)	14.1 (11,512)

a Data source: Office of National Statistics: http://www.statistics.gov.uk/statbase/Product.asp?vlnk=601. *Accessed August 2011*.

b Estimated by the Yorkshire and Humberside Public Health Observatory model, including diagnosed and undiagnosed diabetes in the community. http://www.yhpho.org.uk/resource/view.aspx?RID=78382 *Accessed August 2011*.

**Figure 1:**
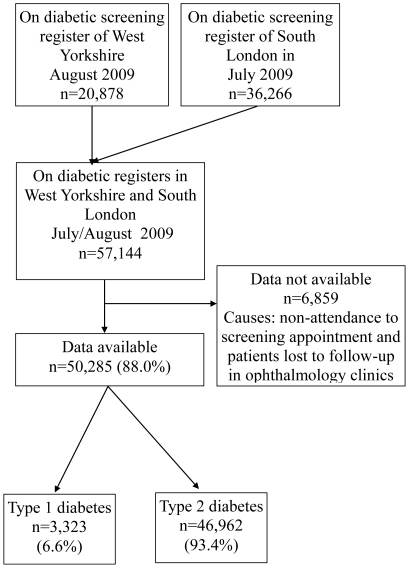
Flow chart of participants in the study.

Data on diabetic retinopathy were available in 50,285 (88.0%) people (figure 1). Table 3 shows the characteristics of the people with data on diabetic retinopathy. The mean age of the population with type 1 diabetes was 39.4±16.3 years while that of type 2 was 63.6±13.3 years. Approximately 63% of people with type 2 diabetes were aged 60 years and over. A greater proportion of people with type 2 diabetes were of non-white origin compared to type 1 diabetes.

**Table 3:** Characteristics of the study population.

	Type 1 diabetesN = 3,323	Type 2 diabetesN = 46,962
Mean age (SD)	39.4 (16.3)	63.6 (13.3)

	*N(%)*	*N(%)*
Age <20	326 (9.8)	23 (0.1)
Age 20–29	748 (22.5)	134 (0.3)
Age 30–39	755 (22.7)	1634 (3.5)
Age 40–49	680 (20.5)	5779 (12.3)
Age 50–59	382 (11.5)	9802 (20.9)
Age 60–69	235 (7.1)	12352 (26.3)
Age 70–79	152 (4.6)	11843 (25.2)
Age 80+	44 (1.3)	5366 (11.4)
Male	1764 (53.1)	24842 (52.9)
White Europeans	2628 (79.2)	30352 (64.7)
African/Afro-Caribbean	344 (10.4)	8023 (17.1)
South Asian	120 (3.6)	3397 (7.2)
Mixed race	105 (3.2)	2577 (5.5)
Other	123 (3.7)	2587 (5.5)

The prevalence of diabetic retinopathy in the three ethnic groups is shown in tables 4 and 5. In type 1 diabetes, there were some differences between the ethnic groups but these differences were largely not statistically significant due to the small number of people in some groups. In type 2 diabetes, the prevalence of any DR was significantly higher in the African/Afro-Caribbean group (52.4%, 95% confidence intervals (CI)51.2% to 53.6%) and South Asians (42.3%, 95% CI 40.3% to 44.2%) compared to white Europeans (38%, 95% CI 37.4% to 38.5%). Clinically significant macular oedema was nearly three times more common in African/Afro-Caribbean (10.1%, 95% CI 9.4% to 10.8%) and twice as prevalent in South Asians (7.1%, 95% CI 6.0% to 8.1%) compared to white Europeans 3.7% (95% CI 3.5%to 3.9%) . Sight threatening diabetic retinopathy was also twice as prevalent in the non-white ethnic groups (table 5).

**Table 4:** Prevalence of retinopathy in people with type 1 diabetes.

Ethnic group	Prevalence: N (%)	Age-standardised prevalence^a^: % (95% CI)
**Any diabetic retinopathy (R1 or R2 or R3)**		
White Europeans	1446 (55.0)	55.0 (53.2,56.9)
African/Afro-Caribbean	154 (44.8)	42.8 (37.3, 48.3)
South Asian	64 (53.3)	54.0 (44.8, 63.2)
**Any maculopathy (M1)**		
White Europeans	371 (14.1)	14.1 (12.8,15.4)
African/Afro-Caribbean	47 (13.7)	13.1 (9.4,16.8)
South Asian	17 (14.2)	16.6 (10.0, 23.2)
**CSMO (M1P1)**		
White Europeans	171 (6.5)	6.5 (5.6, 7.4)
African/Afro-Caribbean	35 (10.2)	10.0 (6.7,13.3)
South Asian	12 (10.0)	11.2 (5.4,16.9)
**STDR (R2 or R3 or M1P1)**		
White Europeans	318 (12.1)	12.1 (10.9,13.3)
African/Afro-Caribbean	53 (15.4)	15.9 (11.8, 20.0)
South Asian	19 (15.8)	17.5 (10.6, 24.3)

a Standardised to the age-structure of the white European population;

Number (missing data on age): White Europeans n = 2,628 (0) African/Afro-Caribbean n = 344 (1), South Asian n = 120 (0).

CSMO- clinically significant macular oedema; M1- maculopathy P1- macular laser; STDR- sight threatening diabetic retinopathy; R1- mild to moderate non-proliferative diabetic retinopathy; R2- pre-proliferative diabetic retinopathy; R3- Proliferative diabetic retinopathy

**Table 5:** Prevalence of diabetic retinopathy in people with type 2 diabetes.

Ethnic group	Prevalence: N (%)	Age-standardised prevalence^a^: % (95% CI)
**Any diabetic retinopathy (R1 or R2 or R3)**		
White Europeans	11538 (38.0)	38.0 (37.4, 38.5)
African/Afro-Caribbean	4117 (51.3)	52.4(51.2, 53.6)
South Asian	1350 (39.7)	42.3 (40.3, 44.2)
**Any maculopathy (M1)**		
White Europeans	2249 (7.4)	7.3 (7.1, 7.6)
African/Afro-Caribbean	1037 (12.9)	14.0 (13.2,14.8)
South Asian	396 (11.7)	12.6 (11.2,13.9)
**CSMO (M1P1)**		
White Europeans	1127 (3.7)	3.7(3.5, 3.9)
African/Afro-Caribbean	720 (9.0)	10.1(9.4,10.8)
South Asian	211 (6.2)	7.1(6.0, 8.1)
**STDR (R2 or R3 or M1P1)**		
White Europeans	1680 (5.5)	5.5 (5.3, 5.8)
African/Afro-Caribbean	827 (10.3)	11.5 (10.7,12.3)
South Asian	314 (9.2)	10.3(9.0, 11.5)

a Standardised to the age-structure of the Caucasian population;

Number (missing data on age): White Europeans n = 30,350 (20) African/Afro-Caribbean n = 8,023 (0) South Asian n = 3,397 (8).

CSMO- clinically significant macular oedema; M1- maculopathy P1- macular laser; STDR- sight threatening diabetic retinopathy; R1- mild to moderate non-proliferative diabetic retinopathy; R2- pre-proliferative diabetic retinopathy; R3- Proliferative diabetic retinopathy.

Logistic regression analyses showed that the risk of diabetic retinopathy, sight threatening diabetic retinopathy and clinically significant macular oedema increased with increasing age. Women and people with type 2 diabetes had a lower risk of diabetic retinopathy compared to men and people with type 1 diabetes respectively. Minority ethnic groups (both South Asians and African/Afro-Caribbeans) had increased odds of having retinopathy compared to their white counterparts. There were differences between the locations in prevalence of diabetic retinopathy but these were not consistent (table 6).

**Table 6:** Logistic regression analyses.

Adjusted odds ratio* (95% CI)	Any retinopathyR1, R2 or R3(n = 20,344)	Any proliferative diabetic retinopathyR2 or R3(n = 2,195)	CSMOM1P1(n = 2,446)	STDRR2 or R3 or M1P1(n = 3,426)
Age (per year age)	1.007 (1.006, 1.008)	1.005 (1.002, 1.009)	1.019 (1.016,1.022)	1.012 (1.099,1.015)
Men	1	1	1	1
Women	0.93 (0.90,0.97)	0.77 (0.71,0.84)	0.91 (0.84,0.99)	0.84 (0.78,0.90)
West Yorkshire	1	1	1	1
South East London	0.91 (0.87, 0.94)	0.69 (0.63, 0.77)	1.79 (1.61, 1.99)	1.04 (0.96,1.13)
Type I diabetes	1	1	1	1
Type 2 diabetes	0.47 (0.43, 0.51)	0.32 (0.27, 0.37)	0.42 (0.35, 0.49)	0.35 (0.31, 0.40)
White Europeans	1	1	1	1
African/Afro-Caribbeans	1.79 (1.70, 1.89)	1.61 (1.42, 1.82)	2.12 (1.91, 2.35)	1.99 (1.81,2.18)
South Asians	1.10 (1.02, 1.18)	1.52 (1.31, 1.77)	1.98 (1.71, 2.30)	1.82 (1.61, 2.06)
Other	0.75 (0.70, 0.80)	0.59 (0.48, 0.72)	0.68 (0.57, 0.81)	0.68 (0.58, 0.79)

* Adjusted for all factors on the table.

CSMO- clinically significant macular oedema; R1- Mild to moderate non-proliferative diabetic retinopathy; R2- Severe non-proliferative diabetic retinopathy; R3- Proliferative diabetic retinopathy; M1P1- laser treated diabetic maculopathy; STDR-sight threatening diabetic retinopathy.

Data stratified by age shows that prevalence of any DR increases proportionately with age for each racial group and the prevalence is higher in the minority ethnic groups at all ages. However, no age related increase in prevalence for sight threatening diabetic retinopathy and clinically significant macular oedema were observed in any of the ethnic groups although the prevalence was higher in African/Afro-Caribbeans and South Asians (data not shown).

## Discussion

The DRIVE UK study is the largest cross-sectional study on the prevalence of DR in the various ethnic groups with diabetes in the UK. The study shows that the prevalence of any retinopathy in type 2 diabetes is highest in people of African/Afro-Caribbean descent compared to South Asians or white Europeans. Both South Asians and African/Afro-Caribbeans have about double the prevalence of clinically significant macular oedema and sight threatening diabetic retinopathy compared to the white Europeans. There were differences in diabetic retinopathy between the ethnic groups in type 1 diabetes but these are not statistically significant due to the small number of people in the study sample.

This study consisted of people with diabetes in two community-based diabetic retinopathy screening programmes, one representing the inner city population cohort in UK (South East London) and the other reflecting regions within UK with pockets of minor ethnic groups (West Yorkshire). Of the 57,144 people diagnosed with diabetes in the two regions, data on DR was available in 50,285 (88.0%) which is comparable to the response rates of other population-based studies.

The prevalence of diagnosed diabetes in this study is 4.1% with similar rates of diagnosed diabetes between the three ethnic groups. Although the public health model in UK [2] shows a disproportionate burden of diabetes in South Asians and African/Afro-Caribbeans, this study suggests that undiagnosed diabetes and/or uptake of retinal screening remain an issue especially in the minor ethnic groups in the UK.

The overall prevalence of diabetic retinopathy in type 1 diabetes was 53.1%, clinically significant macular oedema of 8.9% and sight threatening diabetic retinopathy of 14.4%. Although there is no historical comparative data in the UK, it is reassuring to note that the prevalence in this population is very similar to that observed in the Nordic countries (41.8% diabetic retinopathy and 12.1% sight threatening diabetic retinopathy) [10] where there is overwhelming evidence of a decline in the incidence of sight threatening diabetic retinopathy compared to reports published two decades ago [11], [12].

In type 2 diabetes, the overall prevalence of diabetic retinopathy was 39.5%, of clinically significant macular oedema was 4.7% and sight threatening diabetic retinopathy was 8.3% in keeping with estimates generated by other contemporary studies in the US and UK [4]–[5]; [13]–[14]. The prevalence of diabetic retinopathy in UK has remained constant over last two decades despite the global increase in the prevalence of diabetes [15], the changing population composition and the improved diagnostic criteria and examination techniques.

Studies conducted before the UKPDS era that compared the prevalence of diabetic retinopathy between African/Afro-Caribbean and white Europeans in the UK did not reveal significant differences between the two groups [16], [17]. However, contemporary comparative data in the US show higher rates in people on African descent [18]–[21]. Both the ARIC [19] and MESA [5] studies noted that these differences were negated when other risk factors for diabetic retinopathy were included in the regression model. This study did not assess other risk factors of diabetic retinopathy such as duration of diabetes, control of hyperglycemia, hypertension and smoking status that may further help define the differences in prevalence between these ethnic groups.

The strength of our study is the use of a substantial dataset of a representative multiethnic population with physician diagnosed diabetes and the use of standard national quality-assurance protocols for post-mydriatic 2-field high-resolution digital photographs and grading of diabetic retinopathy.

One limitation of this study is the use of a grading system that is not universally used in epidemiologic studies making it difficult to compare the prevalence of clinically significant macular oedema. However, the prevalence of diabetic retinopathy in type 2 diabetes in the white population in this study (38%) was similar to that found other recent studies in the world that used either the interim or final ETDRS levels. Secondly, this study is limited to those who attend screening and treatment for DR so it likely that the rates may be an underestimation. Additionally, we have not considered retinopathy rates in people with undiagnosed diabetes. Although we have adjusted for age, gender, type of diabetes, ethnicity and region, we did not assess other traditional risk factors of diabetic retinopathy such as glycemic control, blood pressure and duration of diabetes as it was beyond the scope of this study. It would be useful to observe whether ethnicity remains an independent risk factor, as reports on this aspect are conflicting. Further studies should also focus on other potential reasons for these ethnic differences such as differential susceptibility to risk factors, genetic and behavioural variations, later diagnosis of diabetes and differences in access to healthcare including rates of uptake/compliance with evidence based treatments.
